# Social Support and Well-Being Among Community-Dwelling Older Adults: The Mediating Role of Psychological Resilience

**DOI:** 10.3390/healthcare14142215

**Published:** 2026-07-21

**Authors:** Hang Cheng, Xinyu Dou, Qihui Gan, Junhui Xiao, Xiuchan Song, Jin Huang, Jinzhi Huang, Qikang Chen, Yuxi Liu

**Affiliations:** 1 Shunde Women and Children’s Hospital of Guangdong Medical University, Foshan 528300, China; chenghang0626@gdmu.edu.cn (H.C.); huangjzgd@163.com (J.H.); 2 School of Humanities and Management, Guangdong Medical University, Dongguan 523808, China; douxy12138@outlook.com (X.D.); ganqihui@gdmu.edu.cn (Q.G.); xiaojunhui@gdmu.edu.cn (J.X.); 3 Dongguan Hong Mei Hospital, Dongguan 523160, China; songxiuchan@gmail.com; 4 Dongguan Shijie Town Community Health Service Center, Dongguan 523290, China; hj_dgcdc@126.com; 5 Institute of Health Law and Policy, Guangdong Medical University, Dongguan 523808, China

**Keywords:** social support, psychological resilience, well-being, ageing population, mediation mechanism

## Abstract

**Background/Objective**: With the rapid ageing of the global population, promoting mental health and well-being among older adults has become a key priority for healthcare systems. Social support is widely recognized as an important social determinant of health; however, the underlying psychological mechanisms through which it influences well-being in community-dwelling older adults remain insufficiently understood. This study aimed to examine the association between social support and subjective well-being among community-dwelling older adults in urban China and to determine whether psychological resilience mediates this relationship after adjusting for key sociodemographic factors. **Methods:** A cross-sectional study was conducted between May and July 2023 among 1227 older adults aged ≥ 65 years in three urban communities in Dongguan, China. Stratified cluster sampling was applied. Social support was assessed using the Lubben Social Network Scale (LSNS-6), psychological resilience using the Connor–Davidson Resilience Scale (CD-RISC-10), and well-being using the Elderly Well-Being Scale (EWBS). Pearson correlation and mediation analyses (PROCESS Model 4 with bootstrap resampling) were performed to examine the relationships among variables, adjusting for key sociodemographic factors. **Results**: A significant positive correlation emerged between social support and well-being (r = 0.410, *p* < 0.01), with psychological resilience exhibiting an even stronger correlation with well-being (r = 0.573, *p* < 0.01). Mediation analyses showed that psychological resilience partially mediated the social support–well-being relationship (indirect effect = 0.324, 95% CI: 0.262–0.391), explaining 38.5% of the total effect. Higher educational attainment and stable economic security (e.g., pension coverage) were significantly associated with better well-being outcomes. **Conclusions**: These findings suggest that social support is associated with better well-being both directly and indirectly through psychological resilience. From a healthcare perspective, community-based interventions that strengthen social networks and improve psychological resilience may represent effective strategies for promoting healthy ageing. Integrating psychosocial support and resilience-building programs into primary care and community health services could contribute to improving mental health and quality of life in ageing populations.

## 1. Introduction

Population ageing has emerged as a major global public health challenge, placing increasing pressure on healthcare systems worldwide [1]. As life expectancy continues to increase, healthcare systems are facing a growing demand for long-term care, mental health services, and community-based support for older adults. Promoting healthy ageing and improving the quality of life of older populations have therefore become key priorities in contemporary healthcare policy and practice [2]. In this context, there is an increasing recognition that health in older age extends beyond the absence of disease and should encompass psychological and social well-being.

Subjective well-being (SWB) is widely recognized as an important indicator of mental health and quality of life in older adults. Evidence suggests that higher levels of SWB are associated with better physical health, improved functional ability, reduced healthcare utilization, and lower mortality risk [3]. Conversely, poor SWB may increase vulnerability to chronic diseases, social isolation, and functional decline, thereby placing additional burdens on healthcare systems [3,4]. From a healthcare perspective, identifying modifiable determinants of well-being is essential for designing effective preventive strategies and community-based interventions.

Social support has been consistently identified as a key social determinant of health in ageing populations [5]. It includes emotional support, material assistance, and social integration, all of which contribute to buffering stress and enhancing coping capacity [6]. In the context of healthcare, strong social support networks have been associated with improved treatment adherence, reduced hospitalization rates, and better mental health outcomes among older adults [7]. Despite extensive evidence supporting a positive association between social support and well-being, most existing studies have focused primarily on direct relationships, with limited attention to the underlying mechanisms through which social support exerts its influence [8].

Psychological resilience, defined as the capacity to adapt positively in the face of adversity, has emerged as an important protective factor in ageing research [9]. It plays a crucial role in enabling older adults to cope with health-related challenges, life transitions, and social changes. From a healthcare intervention perspective, resilience is particularly valuable because it is modifiable and can be enhanced through targeted psychosocial programs, such as cognitive–behavioral interventions, social engagement activities, and community-based support services [10]. Increasing evidence suggests that social support may strengthen psychological resilience, which in turn contributes to improved well-being [8]. However, empirical studies examining this mediating pathway remain limited, particularly in rapidly ageing urban populations.

The theoretical interpretation of resilience in later life can also be enriched by the framework of successful aging. Recent studies have shown that social support and resilience are associated with prosocial behaviors [11], suggesting that external social resources and internal adaptive capacity may jointly support positive social functioning. Emotional intelligence and perceived social support have been found to be related to subjective happiness and life satisfaction [12]. Evidence from older adult also suggests that emotional intelligence may predict prosocial behaviors [13], further highlighting the importance of emotional regulation and prosocial engagement in later-life psychosocial functioning.

In addition, social and economic context may shape both access to support and the ability to benefit from it. Socioeconomic determinants such as educational attainment and economic security play a critical role in shaping health inequalities in later life [14]. Older adults with higher education levels and stable income sources often have better access to healthcare resources, stronger social networks, and greater capacity to maintain psychological well-being [14]. In contrast, socioeconomic disadvantage may restrict opportunities for social participation and reduce the capacity to transform available support into psychological resilience and well-being [6]. Therefore, socioeconomic factors should not be viewed only as control variables, but also as contextual conditions that shape the operation of psychosocial resources. Understanding these relationships is essential for developing equitable and targeted healthcare strategies for ageing populations. Understanding how social support translates into better well-being is essential for designing effective community-based interventions.

Although previous research has provided valuable insights into the relationships among social support, psychological resilience, and well-being, several gaps remain. First, previous studies have mainly examined whether social support is associated with well-being, but less is known about the psychological processes through which social support contributes to well-being in later life [14,15]. Second, psychological resilience may be an important mechanism because it reflects older adults’ capacity to adapt to health-related, social, and emotional challenges; however, its mediating role has not been adequately tested among community-dwelling older adults [16,17]. Third, evidence from rapidly urbanizing Chinese communities remains limited, despite the increasing need for community-based healthcare strategies in response to population ageing [18]. Finally, few studies have considered psychosocial resources and socioeconomic characteristics within the same analytical framework [19,20].

To address these gaps, the present study aims to investigate the relationship between social support and well-being among community-dwelling older adults, with a particular focus on the mediating role of psychological resilience. Using data from older adults in urban communities in Dongguan, China, we construct and test a mediation model to examine the pathways through which social support influences well-being. In addition, we explore the role of key sociodemographic factors, including educational attainment and economic security, in shaping well-being outcomes.

While the hypothesized mediation model provides a useful starting point, we acknowledge that the relationship among social support, resilience, and well-being is likely more dynamic and context-dependent. Recent theoretical advances suggest that meaning in life should be conceptualized as a dependent variable in psychosocial models of coping and well-being rather than as a mediating mechanism [21]; moreover, stress exposure [22], social support types [23], and bidirectional influences [24] may shape these pathways. Although our cross-sectional design precludes testing all these nuances, we explicitly discuss these considerations as critical directions for future research and as qualifications of our findings.

By integrating psychosocial and socioeconomic perspectives, this study seeks to provide empirical evidence to inform community-based healthcare interventions and policy development. The findings are expected to contribute to the design of integrated strategies that combine social support enhancement and resilience-building approaches, ultimately promoting healthy ageing and improving mental health and quality of life among older populations.

## 2. Materials and Methods

### 2.1. Study Design and Participants

This cross-sectional study was conducted between May and July 2023 in three districts of Dongguan, China, namely Tangxia, Songshan Lake, and Liaobu. A stratified cluster sampling method was employed to recruit community-dwelling older adults.

Eligible participants were required to meet the following inclusion criteria: (1) aged 65 years or older; (2) cognitively intact with the ability to communicate effectively; and (3) willingness to participate and provision of written informed consent. Individuals with severe cognitive impairment or communication difficulties were excluded.

A total of 1344 questionnaires were distributed through face-to-face surveys administered by trained investigators. Data were independently entered by two researchers to ensure accuracy, and invalid or incomplete responses were excluded. Ultimately, 1227 valid questionnaires were included in the final analysis, yielding a response rate of 91.30%.

This study was approved by the Institutional Review Board of the Affiliated Hospital of Guangdong Medical University. All procedures were conducted in accordance with the Declaration of Helsinki, and written informed consent was obtained from all participants.

### 2.2. Measures

#### 2.2.1. Sociodemographic Characteristics

A self-designed questionnaire was used to collect participants’ sociodemographic information, including gender, age, marital status, educational level, and primary source of income.

#### 2.2.2. Elderly Well-Being Scale (EWBS)

The Elderly Well-Being Scale (EWBS), a culturally specific instrument developed and validated in Chinese populations, was used to assess subjective well-being [25]. It consists of 21 items across four domains: material conditions, health status, social belonging, and spiritual fulfillment. Each item is rated on a 5-point Likert scale ranging from 1 (very dissatisfied) to 5 (very satisfied). The total score ranges from 0 to 105 and is subsequently standardized, with higher scores indicating greater well-being. Although an internationally standardized version is not currently available, the EWBS has been widely used in Chinese ageing research and has demonstrated satisfactory psychometric properties. In this study, the internal consistency was acceptable, with a Cronbach’ s α of 0.838.

#### 2.2.3. Lubben Social Network Scale (LSNS-6)

Social support was measured using the 6-item Lubben Social Network Scale (LSNS-6), which evaluates social engagement through family and friend networks. Each item is scored on a 6-point scale (0–5), with total scores ranging from 0 to 30. Higher scores indicate stronger social networks, while scores below 12 suggest a risk of social isolation. The LSNS-6 is widely used in geriatric populations and has demonstrated good psychometric properties [26]. In this study, Cronbach’s α was 0.808.

#### 2.2.4. Connor–Davidson Resilience Scale 10 (CD-RISC-10)

Psychological resilience was assessed using the 10-item Connor–Davidson Resilience Scale (CD-RISC-10). Each item is rated on a 5-point Likert scale ranging from 0 (never) to 4 (always), with total scores ranging from 0 to 40. Higher scores reflect greater resilience, representing stronger adaptability to stress and adversity. The CD-RISC-10 has been validated in various populations, including older adults [27]. In this study, the scale demonstrated high internal consistency (Cronbach’ s α = 0.893).

### 2.3. Quality Control

To ensure data quality, all investigators underwent standardized training prior to data collection, including instruction on epidemiological survey methods, questionnaire administration, and ethical considerations. A pilot training session was conducted to familiarize investigators with the study protocol and ensure consistency in data collection procedures. Mock interviews and field simulations were performed to enhance reliability. During data collection, on-site supervision and verification were implemented to minimize bias and ensure completeness of responses.

### 2.4. Statistical Analysis

Data were analyzed using SPSS version 26.0. Descriptive statistics are used to summarize participant characteristics, with categorical variables presented as frequencies and percentages, and continuous variables expressed as mean ± standard deviation (x ± s). Group differences were assessed using independent samples t-tests (for two groups) or one-way analysis of variance (ANOVA) (for multiple groups). Pearson correlation analysis was conducted to examine the relationships among social support, psychological resilience, and well-being.

To test the mediating effect of psychological resilience, the PROCESS macro (Model 4) developed by Hayes was applied with bootstrap resampling (n = 5000). A mediating effect was considered statistically significant if the 95% confidence interval (CI) of the indirect effect did not include zero. The proportion of mediation was calculated to assess the relative contribution of the indirect effect.

In the mediation analysis, sociodemographic variables that showed significant associations with SWB in univariate analyses or were theoretically relevant were included as covariates. These included age, sex, marital status, educational level, and source of income. Categorical variables were entered into the regression models using dummy coding, with the lowest-risk or most common category used as the reference group. Before conducting the regression and mediation analyses, model assumptions were examined. Multicollinearity was assessed using variance inflation factors, residual plots were inspected to evaluate linearity and homoscedasticity, and the distribution of residuals was checked to assess approximate normality. No serious violations of regression assumptions were identified.

A two-tailed *p* value < 0.05 was considered statistically significant.

## 3. Results

### 3.1. Participant Characteristics

A total of 1227 older adults were included in the analysis. The mean age of participants was 68.5 years (SD = 3.4), and 59.80% were female. Most participants were married (80.10%), and 53.20% had completed primary education or above. Approximately 89.30% reported having stable economic security (e.g., pension coverage, social assistance and financial support from their adult children).

Significant differences in subjective well-being (SWB) were observed across gender, education levels and economic status (*p* < 0.05), with higher SWB scores among male participants, higher educational attainment and stable income sources (Table 1).

### 3.2. Correlations Analysis Among Social Support, Psychological Resilience, and SWB

**Figure 1:**
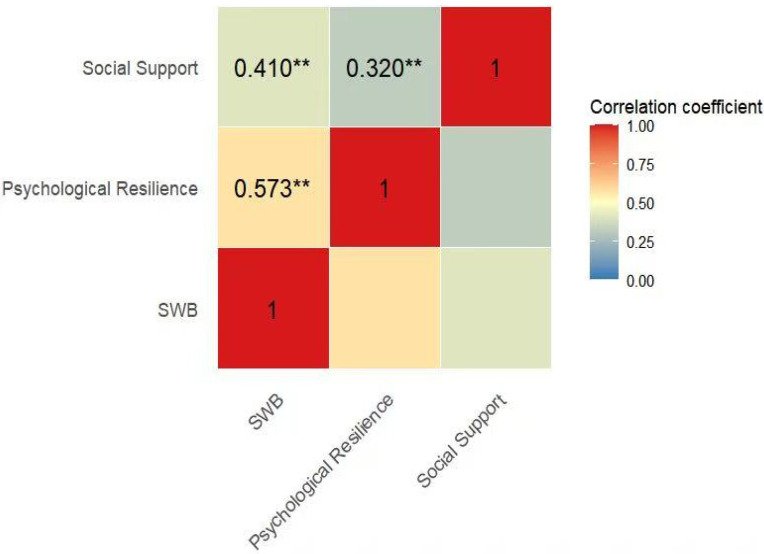
Correlation heat map of social support, psychological resilience, and subjective well-being. Note: ** *p* < 0.01 (two-tailed).

Pearson correlation analysis showed that social support was positively associated with well-being (r = 0.410, *p* < 0.01). Psychological resilience was also positively correlated with well-being (r = 0.573, *p* < 0.01) and with social support (r = 0.320, *p* < 0.01), indicating that higher levels of social support were associated with stronger resilience and better well-being. Table 2 presents the detailed results. To provide a clearer visualization of the correlations among social support, psychological resilience, and SWB, a correlation heat map was generated using R and is presented in Figure 1.

### 3.3. Regression Analysis of Psychological Resilience’s Mediating Role in the Relationship Between Social Support and SWB

A stepwise regression model was utilised to analyse psychological resilience’s mediating effect and gradually clarify the associations and hypothesized pathways among the study variables. In the first step, for each 1-unit increase in social support, psychological resilience increased by 0.365 units (β = 0.365, *p* < 0.001), indicating that external support enhances psychological resilience. In the next step, social support’s direct effect on SWB was found to be significant (β = 0.518, *p* < 0.001). After psychological resilience was included in the model, psychological resilience showed a significant positive association with SWB (β = 0.888, *p* < 0.001), while the indirect pathway from social support to SWB through psychological resilience was further supported by the bootstrap mediation analysis. The mediation analysis suggested a potential indirect pathway linking social support to SWB through psychological resilience. Specifically, higher social support was associated with greater psychological resilience, which in turn was associated with higher SWB. Table 3 presents the detailed results.

### 3.4. Analysing Psychological Resilience’s Mediating Effect in the Relationship Between Social Support and SWB

**Figure 2:**
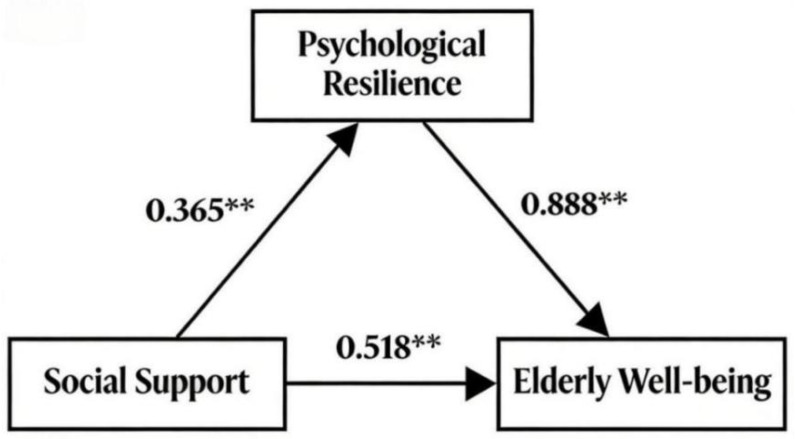
Structural equation model of the mediating effect of resilience on social support and SWB. Note: ** *p* < 0.01 (two-tailed).

The Bootstrap test’s results (Table 4) revealed a significant mediating effect of psychological resilience on the relationship between social support and SWB. Social support’s indirect effect value on SWB was 0.324 (95% CI: 0.262–0.391), accounting for 38.5% of the total effect. The direct effect value was 0.518 (95% CI: 0.422–0.613), accounting for 61.5% of the total effect. These results suggest that psychological resilience played a partial mediating role in the relationship between social support and SWB, implying that social support not only directly promotes SWB but also exerts an indirect effect by enhancing psychological resilience. This result supported the theoretical pathway of ‘social support → psychological resilience → SWB’, as illustrated in Figure 2.

## 4. Discussion

This study examined the association between social support and subjective well-being (SWB) among community-dwelling older adults, with a particular focus on the mediating role of psychological resilience. The findings indicate that social support is positively associated with SWB. Moreover, psychological resilience partially mediates this relationship, suggesting that social support may enhance well-being both directly and indirectly by strengthening individuals’ adaptive capacity. In addition, socioeconomic factors, particularly educational attainment and economic security, were significantly associated with SWB outcomes.

The positive association between social support and SWB observed in this study is consistent with a substantial body of literature demonstrating the protective role of social support in older adults’ mental health. Social support has been widely recognized as a buffering factor that reduces the negative impact of stress and enhances psychological resilience [28]. Moreover, it may mitigate social isolation and promote emotional stability through both direct and indirect pathways, thereby contributing to improved SWB [29,30]. Empirical studies focusing on older populations further confirm that higher levels of perceived social support are associated with lower depressive symptoms and better life satisfaction [31,32]. Additionally, recent meta-analytic evidence highlights social support as a clinically relevant protective factor against depression, particularly among older adults and caregivers [33]. These findings collectively support the notion that social support plays a crucial role in maintaining mental health and SWB in aging populations.

Importantly, the present study extends previous research by empirically supporting the mediating role of psychological resilience in the relationship between social support and SWB. While earlier studies have predominantly focused on the direct association between social support and mental health outcomes, emerging evidence suggests that internal psychological resources, particularly resilience, play a crucial intermediary role [34,35]. Psychological resilience may enable individuals to better utilize available social resources, adapt to stress, and maintain emotional stability, thereby facilitating the translation of social support into enhanced well-being [36,37]. Recent empirical studies have demonstrated that resilience significantly mediates the relationship between social support and mental health outcomes, including life satisfaction and depressive symptoms [38]. This highlights the importance of considering both external social resources and internal psychological capacities in understanding mental health outcomes in ageing populations.

The mediating role of psychological resilience should not be interpreted only as a stress-coping mechanism. For older adults, resilience may also reflect the capacity to maintain meaning, purpose, and identity despite age-related transitions [39,40]. Social support from family members, friends, and community networks may enhance well-being by reinforcing older adults’ sense of being valued and socially needed [41,42]. Therefore, future studies may examine meaning in life as an additional dependent variable, or as a parallel indicator of well-being, rather than as a mediator [21].

Furthermore, the observed influence of socioeconomic factors aligns with existing evidence on health inequalities in later life. Socioeconomic status (SES), particularly educational attainment and financial stability, has been widely recognized as a key determinant of health and SWB [43,44]. Individuals with higher levels of education and more stable economic resources are more likely to access healthcare services, engage in social activities, and adopt effective coping strategies, all of which contribute to better mental health outcomes and higher levels of SWB [45]. Recent studies indicate that socioeconomic advantages are associated with improved successful aging outcomes and greater psychological resources, while lower SES is linked to reduced access to care and poorer health trajectories in older populations [46,47]. These findings highlight the critical role of socioeconomic resources in shaping health trajectories in ageing populations and underscore the importance of addressing social inequalities in promoting healthy ageing.

Although the present findings are consistent with previous research, their contribution should be interpreted cautiously. The study confirms an important psychosocial association in a large community sample, but it does not fully explain the dynamic process through which social support, resilience, and well-being may influence each other over time. Resilience in later life should not be understood only as stress coping; it may also involve emotional regulation, meaning maintenance, social engagement, and prosocial orientation. Older adults with stronger emotional and social resources may be more likely to maintain supportive relationships, participate in community life, and preserve a sense of personal and social value. Future research should therefore examine emotional intelligence, prosocial behaviors, and different forms of social support as potential mediators or moderators in the relationship among social support, resilience, and SWB, while meaning in life should be examined as an additional dependent variable or as a parallel indicator of well-being.

From a healthcare perspective, these findings have important implications for the development of community-based and primary care interventions targeting older adults. First, enhancing social support networks should be considered a key strategy for promoting SWB, as recent evidence suggests that structured social participation programs, including group activities and peer support initiatives, are effective in improving mental health and reducing loneliness among older populations [15,48]. Second, psychological resilience represents a modifiable and clinically relevant target for intervention. Integrating resilience-building approaches—such as cognitive-behavioral strategies, mental health education, and social engagement programs—into community healthcare services may improve older adults’ ability to cope with health-related and social challenges [49,50]. Third, the significant role of socioeconomic factors suggests that healthcare interventions should be complemented by broader social and policy measures. Recent studies indicate that improving financial security and access to lifelong learning opportunities can reduce health inequalities and promote more equitable well-being outcomes in ageing populations [44,51]. These findings highlight the importance of integrated, multi-level strategies that combine healthcare, community support, and social policy to promote healthy ageing.

At the policy level, the findings support the development of integrated healthcare strategies that combine psychosocial support with structural interventions [2,52]. In rapidly ageing societies, particularly in urban China, strengthening community-based healthcare systems is essential to address the growing demand for mental health and supportive services [53,54]. Policies that promote age-friendly communities, expand access to community health services, and integrate mental health care into primary care settings may enhance both social support and psychological resilience among older adults [55,56]. Such approaches are consistent with global healthy ageing frameworks and may contribute to reducing healthcare burden in the long term [2,57].

This study has several strengths, including a relatively large sample of community-dwelling older adults, use of validated instruments (LSNS-6, CD-RISC-10, EWBS), and a mediation framework that integrates psychosocial and socioeconomic factors. However, several limitations should be acknowledged. First, the cross-sectional design precludes causal inference. Although the mediation model was theoretically specified from social support to resilience to well-being, reverse or reciprocal relationships are plausible: older adults with higher well-being or resilience may be more proactive in seeking and maintaining social support. Longitudinal cross-lagged panel designs are needed to test causality and bidirectional effects. Second, all variables were measured using self-reported questionnaires, which may introduce recall bias, common method bias, and social desirability bias. Third, the LSNS-6 captures mainly family and friend networks but does not fully distinguish emotional, instrumental, informational, perceived, and received support. Fourth, the study was conducted in urban communities in Dongguan, China, which may limit generalizability to rural areas, other regions, or different sociocultural contexts. Finally, some potentially important variables—such as stress exposure, meaning in life, emotional intelligence, prosocial behavior, chronic disease status, functional ability, and access to community health services—were not fully examined. Future intervention studies that randomly assign older adults to resilience-training or social-enhancement programs could provide actionable evidence for healthcare systems.

## 5. Conclusions

This study provides evidence that social support was positively associated with subjective well-being among community-dwelling older adults, and psychological resilience appeared to play a partial mediating role in this association. By identifying psychological resilience as a key mediating pathway, the findings offer new insights into how social and psychological resources interact to influence mental health in ageing populations. These results have important implications for healthcare practice and policy. Strengthening social support networks and implementing resilience-based interventions within community and primary care settings may represent effective approaches to promoting healthy ageing. In addition, addressing socioeconomic disparities remains essential for achieving equitable health outcomes. Taken together, this study supports the development of integrated, person-centered healthcare strategies aimed at improving mental well-being and quality of life among older adults.

## Figures and Tables

**Table 1:** Univariate Analysis of Elderly Subjective Well-Being (SWB) Scores.

Variables	Categories	n (%)	SWB Score ($\bar{x}\pm s$)	t/F Value	Effect Size	*p*-Value
Age	60–69	677 (55.18)	67.87 ± 12.70	5.26	η^2^ = 0.008	0.063
	70–79	447 (36.42)	64.60 ± 13.94			
	≥80	103 (8.40)	62.05 ± 11.28			
Gender	Male	493 (40.20)	67.88 ± 13.82	3.19	d = 0.186	<0.001
	Female	734 (59.80)	65.41 ± 12.96			
Marital status	Unmarried	4 (0.33)	63.99 ± 14.43	5.95	η^2^ = 0.010	0.089
	Married	983 (80.11)	66.20 ± 13.29			
	Widowed	240 (19.56)	63.40 ± 13.36			
Education level	Illiterate	168 (13.70)	59.25 ± 13.82	33.52	η^2^ = 0.099	<0.001
	Primary school	485 (39.50)	63.79 ± 12.77			
	Junior high school	300 (24.40)	68.43 ± 11.61			
	Senior high school	205 (16.70)	72.53 ± 12.49			
	college and above	69 (5.60)	74.37 ± 11.27			
Main source of income	Pension	709 (57.80)	67.75 ± 13.80	8.09	η^2^ = 0.019	<0.001
	Social assistance	35 (2.90)	55.34 ± 13.63			
	Support from adult children	351 (28.60)	65.30 ± 12.10			
	Others	132 (10.80)	60.53 ± 10.84			

Note: Cohen’s d was reported for two-group comparisons, and eta-squared (η^2^) was reported for ANOVA. Statistical significance was set at *p* < 0.05 (two-tailed).

**Table 2:** Correlation Analysis of Social Support, Psychological Resilience and Elderly SWB.

Variables	SWB	Psychological Resilience	Social Support
SWB	1		
Psychological resilience	0.573 **	1	
Social support	0.410 **	0.320 **	1

Note: ** *p* < 0.01 (two-tailed). Correlation coefficients are Pearson’s r.

**Table 3:** Regression Analysis of Social Support, Psychological Resilience, and Elderly Well-Being.

Step	Independent Variable	Dependent Variable	β	SE	t	95% CI	Effect Size	*p*-Value
1	Social support	psychological resilience	0.365	0.031	11.83	[0.304, 0.426]	f^2^ = 0.114	<0.001
2	Social support	SWB	0.518	0.049	10.66	[0.422, 0.614]	f^2^ = 0.202	<0.001
3	Psychological resilience	SWB	0.888	0.043	20.84	[0.804, 0.972]	f^2^ = 0.629	<0.001

Note: β = unstandardized regression coefficient. SE = standard error; CI = confidence interval; statistical significance was set at *p* < 0.05 (two-tailed). All *p*-values shown are < 0.001.

**Table 4:** Mediating Effect of Psychological Resilience Between Social Support and SWB.

Type of Effect	Effect Value	Standard Error	95% CI	Relative Effect Proportion (%)
Direct Effect	0.518	0.049	[0.422, 0.613]	61.5%
Indirect Effect	0.324	0.033	[0.262, 0.391]	38.5%
Total Effect	0.841			

## Data Availability

The data presented in this study are not publicly available due to privacy and ethical restrictions to protect participant confidentiality.
